# Aortic root replacement is superior to isolated aortic valve replacement for aortic regurgitation secondary to Behcet's disease

**DOI:** 10.3389/fcvm.2024.1437208

**Published:** 2024-09-02

**Authors:** Jing-Bin Huang

**Affiliations:** Department of Cardiothoracic Surgery, The People’s Hospital of Guangxi Zhuang Autonomous Region, and Guangxi Academy of Medical Sciences, Nanning, Guangxi, China

**Keywords:** bentall procedure, behcet’s disease, aortic regurgitation, paravalvular leakage, mortality

## Introduction

Aortic regurgitation secondary to Behcet's disease is rare but often fatal. If conventional aortic valve replacement surgery is used to treat aortic valve regurgitation secondary to Behcet's disease, valve leakage and hospitalization mortality are high (1–3). Surgical management of aortic regurgitation secondary to Behcet's disease remains grave challenges for cardiac surgeons.

Behcet's disease is a chronic systemic inflammatory disease with unclear etiology, characterized by recurrent oral ulcers and involvement of other organs including the skin, eyes, joints, blood vessels, brain, and gastrointestinal tract. The typical features of Behcet's disease include vascular injury, neutrophil dysfunction, and abnormal autoimmune response (2–4). Based on current evidence, Behcet's disease is believed to be caused by various environmental factors, such as bacterial or viral infections in genetically susceptible individuals. The natural history of Behcet's disease includes both worsening and remission phases. Although the pathogenesis is not yet clear, many studies have shown the same pathway: an abnormal immunopathological process with many triggering factors, such as inflammation and genetic susceptibility associated with HLA-B51 alleles (3–5). Vasculitis in Behcet's disease is mainly characterized by neutrophils, which affect all layers of blood vessels and vasa vasorum. In the late stage, fibrous thickening and non-specific inflammatory infiltration may occur. Neutrophils in patients with Behcet's disease increase the production of superoxide, enhanced chemotaxis, and excessive production of lysosomal enzymes, indicating excessive activity of neutrophils and ultimately leading to tissue damage (3–5). Cardiovascular complications are relatively rare in Behcet's disease, with only 3% to 6% of patients having cardiac complications, mainly including pericarditis, myocarditis, endocarditis with valve regurgitation, intracardiac thrombosis, myocardial fibrosis, coronary artery aneurysm, and sinus aneurysm. However, cardiovascular complications associated with aortic valve regurgitation and Valsalva sinus aneurysm are the main causes of death in patients with Behcet's disease (1, 5–7).

In patients with Behcet's disease, the aortic annulus and wall are fragile due to inflammation of endothelial tissue. Some people suggest using different surgical techniques to treat aortic regurgitation related to Behcet's disease, in order to reduce the incidence of perivalvular leakage (6). However, there is currently no consensus on which surgical method is more advantageous (1, 5–7).

In patients with Behcet's disease, the aortic valve is damaged, and both the aortic annulus and ascending aorta are affected by the disease. Continuous inflammatory response can lead to fragility of the aortic annulus and ascending aortic wall, ultimately resulting in perivalvular leakage after aortic valve replacement surgery. The inflammatory process of the aortic wall can also lead to the destruction of elastic fibers, resulting in transmural necrosis of the aortic wall in large arteries, leading to the formation of aneurysms. Aortic valve replacement surgery was performed on patients with severe aortic regurgitation associated with Behcet's disease in the past. However, according to reports, the mortality rate and reoperation rate of isolated aortic valve replacement surgery are high (20% to 47.3% and 78% to 100%, respectively) (1, 5–7).

It is reported that overall mortality of surgical management of aortic regurgitation secondary to Behcet's disease was 47.3%, with re-operation rate of 78.9% because of valve detachments (8). According to reports, the incidence of valve detachment in patients undergoing transposition Bentall surgery or valved conduit procedures is much lower than in patients undergoing aortic valve replacement surgery alone. Using the Bentall procedure, no perivalvular leakage was found during the first or second surgery (9).

In the observational study conducted by Hu et al., 38 patients with aortic regurgitation secondary to Behcet's disease underwent surgery, 23 underwent Bentall surgery, and 15 underwent routine aortic valve replacement surgery. 13 cases of perivalvular leakage (34.2%, 13/38) occurred after surgery, with a median time of 5 months, all of whom were in the routine aortic valve replacement surgery group (86.7%, 13/15). During the follow-up period, no patients in the group receiving Bentall surgery experienced perivalvular leakage (1, 9).

In addition to surgical strategies, immunosuppression and steroid therapy have also played a very important role in patients with Behcet's disease. According to reports, immunosuppressive therapy can improve perioperative outcomes and should be initiated before surgery. In patients who received steroid treatment before surgery, the inflammatory response was significantly less severe. In these patients, lymphocyte infiltration was mainly found in the aortic wall and aortic valve. However, in patients who did not receive preoperative steroid treatment, the main infiltrating cells were neutrophils, indicating an acute inflammatory response. Among patients with Behcet's disease undergoing routine aortic valve replacement surgery alone, perivalvular leakage is more common (47%, median interval of 3.5 months). Preoperative immunosuppressive therapy, especially cyclophosphamide combined with glucocorticoids, can reduce the incidence of postoperative perivalvular leakage and improve prognosis (1, 8, 9).

More and more evidence suggests that, supported by recognized surgical principles, aortic root replacement surgery is associated with a good rate of reoperation and an early incidence of perivalvular leakage. During a one-year follow-up, patients who underwent aortic root replacement surgery had a lower risk of reoperation and the incidence of early perivalvular leakage. Perivalvular leakage refers to residual leakage between the artificial valve and the patient's natural valve annulus due to incomplete connection between the artificial valve and the natural valve annulus. In Bentall surgery, self-assembled composite grafts can safely secure the proximal anastomosis of patients with damaged aortic valve annulus, achieving effective proximal anastomosis and extremely low incidence of aortic related reoperation. In aortic root replacement surgery (Bentall surgery), aortic valve leakage generally does not happen. According to reports, improving Bentall surgery can effectively reduce perivalvular leakage associated with aortic regurgitation associated with Behcet's disease (10, 11).

For aortic valve regurgitation secondary to Behcet's disease, paravalvular leakage after routine aortic valve replacement surgery is a complex situation. Due to the fragility and persistent inflammation of the aortic tissue, patients with Behcet's disease who undergo isolated aortic valve replacement surgery have a poor prognosis, with many patients experiencing perivalvular leakage, valve detachment, and pseudoaneurysm after surgery. Compared to individual aortic valve replacement surgery, aortic root replacement surgery may bring better results. Bentall surgery can effectively reduce perivalvular leakage and has better short- and long-term effects. Bentall surgery is superior to simple aortic valve replacement surgery for the treatment of aortic regurgitation secondary to Behcet's disease (1, 12, 13).

Even in patients who do not meet the current diagnostic criteria, severe aortic regurgitation can be the initial manifestation of Behcet's disease. Surgeons should recognize the possibility of cardiovascular Behcet's disease in young patients with unknown etiology of aortic valve regurgitation before or after the first aortic valve replacement surgery, or in cases of perivalvular leakage, artificial valve detachment, and pseudoaneurysm (1, 13).

Quickly and effectively controlling inflammation is crucial for protecting heart function and preventing postoperative complications. It has been proven that the combination of glucocorticoids and immunosuppressants can effectively reduce the occurrence of perivalvular leakage and other complications. However, the use of glucocorticoids and immunosuppressants in surgical treatment may delay wound healing and increase susceptibility to infection. In this case, biologics have good protective effects of glucocorticoids and immunosuppressants, which can effectively and quickly suppress inflammation and promote the wound healing process after cardiac surgery. Perioperative biologic therapy for patients with aortic valve regurgitation caused by Behcet's disease can significantly reduce the incidence of perivalvular leakage and the risk of reoperation, both before and after surgery, and improve the prognosis of aortic valve regurgitation caused by Behcet's disease. Biological agents can reduce the dosage and side effects of glucocorticoids and immunosuppressants, and improve prognosis (14).

There is still controversy over when to start using immunosuppressants. We believe that for patients who will undergo selective surgery and have inactive inflammation, it is recommended to use immunosuppressants before and after surgery. For patients who will undergo emergency surgery, regardless of whether the inflammation is active or inactive, severe aortic valve regurgitation and congestive heart failure that are difficult to control medically may occur. Immunosuppressants should be used as soon as possible after surgery. The combination of perioperative biologics with glucocorticoids and immunosuppressants may be a new strategy for preventing postoperative complications and improving the prognosis of aortic valve regurgitation caused by Behcet's disease (15).

## Limitations and strengths of the opinion

This viewpoint discusses an important issue regarding the best treatment strategy supported by current literature: aortic valve replacement or aortic root replacement? Even though the evidence remains contradictory and more reliable data is needed, aortic root replacement surgery seems to be the most valuable option today.
